# Additional Crypt Phenotypes Lined With High‐Grade Cellular Dysplasia in Protruding Growths in Ulcerative Colitis

**DOI:** 10.1111/apm.70216

**Published:** 2026-05-05

**Authors:** Carlos A. Rubio, Daniel Firmbach, Christian Matek, Michael Vieth, Corinna Lang‐Schwarz

**Affiliations:** ^1^ Department of Oncology and Pathology Karolinska Institute Stockholm Sweden; ^2^ Institute of Pathology Universitätsklinikum Erlangen, Friedrich‐Alexander‐Universität Erlangen‐Nürnberg (FAU) Erlangen Germany

**Keywords:** cellular dysplasia, protruding dysplastic growths, UC‐associated sporadic adenomas, ulcerative colitis (UC)

## Abstract

Two crypt phenotypes (ACP): crypt rings in tandem (CRT) and crypts with lateral budding's (CLB's) were recently found interpolated amidst generally accepted crypt phenotypes (GACP, i.e., tubular, tubulovillous, villous and serrated). The frequency of cases with UC‐PDG, with CRT and with CLB's in GACP and in UC‐associated sporadic adenomas (UC‐aspa) was recorded. The possibility that the presence of ACP could increase histological discrimination between UC‐PDG and UC‐aspa was also explored. Two hundred digitalized biopsy‐cases were investigated: 100 with UC‐PDG and 100 with UC‐aspa. Six GACP and two types of cellular dysplasia: low‐grade (LGD) and high‐grade dysplasia (HGD) were found. CRT and CLB's were present in 66% of the 200 cases. A significantly higher number of cases with villous phenotype wihout or with HGD, with CLB's phenotype without or with HGD, and with tubulovillous phenotype with HGD were found in UC‐PDG than in UC‐asta. Several authors found no significant difference in the histological characteristics between UC‐protruding dysplastic lesions and US‐aspa. This work reveals, however, that the different number of cases with the above‐mentioned histological parameters present in each group permitted to discriminate between UC‐PDG and UC‐asta.

## 1 Introduction

Long‐standing colorectal mucosal inflammation such as in ulcerative colitis (UC) increases the risk for developing preinvasive neoplasia and subsequently colorectal carcinoma (CRC) [1]. The first case of CRC complicating UC was described by Crohn and Rosenburg in 1925 [2], and in 1949, Warren and Sommers reported irregular polypoid lesions with glands lined with dark cells considered as precancer in UC [3].

Based on the finding of atypical squamous cells exfoliated from the uterine cervix, Reagan et al. first launched the concept of cellular dysplasia in 1953 [4], In 1967, Morson and Pang were first in applying the term dysplasia (or precancer) to lesions found in rectal biopsies from patients with UC and CRC [5]. Subsequently, Hultén, Kewenter and Åhrén found villous epithelial proliferations lined with cells showing marked epithelial atypia, and mucin depletion in patients with UC [6]. These villous changes were thought to be precancerous. Cook and Goligher used the term epithelial dysplasia for UC lesions [7], and Lennart Jones et al. also applied the term high‐grade dysplasia in UC [8]. Riddell et al. proposed the following classification of dysplasia in UC in 1983, (i) negative for dysplasia, (ii) indefinite for dysplasia, (iii) probably positive, and (iv) positive for dysplasia [9]. The positive category was subdivided into low‐grade dysplasia (LGD) and high‐grade dysplasia (HGD). For many years the Riddell et al. classification of dysplasia was the cornerstone in diagnosing abnormal cellular changes in UC. In 2004, a Canadian Consensus abridged the classification of UC dysplasia into two categories: LGD and HGD (the latter acronym included terms such as severe dysplastic changes and carcinoma in situ) [10]. In 2015, the SCENIC international endoscopic consensus statement on surveillance and management of dysplasia in inflammatory bowel disease (IBD, i.e., UC and CD), proposed five types of endoscopic morphological scenarios: pedunculated, sessile, superficial elevated, flat, and depressed lesions [11].

The histological classification of generally accepted phenotypes (GACP) present in UC protruding mucosal growths (UC‐PDG) includes tubular, tubulovillous, villous and serrated crypt phenotypes [12]. In 2000 we first reported the occurrence of serrated dysplastic adenomatous growths in IBD [13]; the histological examination of 100 consecutive colectomy specimens with CRC (50 with IBD and 50 without IBD) revealed serrated dysplastic growths juxtaposing CRC in 29% of the IBD specimens, but only 3% of those without IBD [13]. Hence, serrated dysplastic growths juxtaposing CRC evolved more frequently in IBD than in patients with CRC without IBD [13]. In 2007, we studied 96 colectomy specimens with UC and CRC; 73 had UC‐PDG and 31 UC‐associated sporadic adenomas [14]. Out of the 73 UC‐PDG, 48% were villous, and out of the 31 UC‐associated sporadic adenomas, 26% were villous. Thus, villous dysplastic growths evolved more frequently in UC‐PDG than in UC‐associated sporadic adenomas [14]. To histologically discriminate between protruding dysplastic growths in UC (UC‐PDG; growing in areas with inflammation) and UC‐associated sporadic adenomas (growing in areas without inflammation) is clinically crucial as the former carries a higher risk of progression to CRC than the latter [3]. However, Enkelsgjerd et al. [15], Ullman et al. [16], Van Schaik et al. [17] and Wander et al. [18] found that UC‐ dysplastic growths were indistinguishable from UC‐associated sporadic adenomas. Even after applying advanced sequencing techniques, Christakis et al. found a significant overlap in molecular alterations between UC‐protruding dysplasias and UC‐sporadic adenomas [19]. In 1983, Deschner et al. found an unusual crypt phenotype epitomized by nondysplastic crypts with lateral buds in rectal biopsies from patients with UC [20]. In vitro incubation with tritiated thymidine (3H‐TdR) showed that the number of DNA‐synthetizing cells in the lateral buds was higher than in other parts of the crypts in some patients. Deschner claimed that nondysplastic lateral budding's or outpouching's in UC forecasted neoplastic transformation [20]. More recently we reported two alternative dysplastic crypt phenotypes: crypt rings in tandem (CRT) in UC [21] and dysplastic crypts with lateral budding's (CLB's) in sporadic colorectal tubular adenomas [22]. Out of 146 consecutive tubular adenomas of the colorectum, CRT were detected in 40% 21, and CLB's in 26% out of 309 consecutive tubular sporadic colorectal adenomas [22].

The purpose of this study was two folded: (i) to investigate whether interpolated crypt phenotypes also developed in UC‐PDG and in UC‐associated sporadic adenomas (UC‐aspa). If that was the case, and (ii) to explore whether the occurrence of these interpolated crypt phenotypes could help to histologically differentiate between UC‐PDG and UC‐aspa.

## 2 Methods

### 2.1 Biopsy/Cases

The material consists of 200 consecutive protruding dysplastic colonic lesions found in patients with ulcerative colitis (UC). All cases were retrieved from the electronic archive (DC Pathos database), Institute of Pathology, Friedrich‐ Alexander‐Universität, Erlangen‐Nürnberg, Klinikum Bayreuth, Germany (DC Systeme, Nexus, Heiligenhaus, Germany) [23]. Out of the 200 cases, 100 biopsy/cases revealed UC‐PDG (i.e., in mucosal areas with inflammation at endoscopic examination), and 100 cases showed UC‐aspa (i.e., in mucosal areas without inflammation at endoscopic examination). Sections were cut into 4‐μm‐thick sections and stained with hematoxylin–eosin (H&E). The H&E‐stained slides were subsequently scanned and digitalized with a Hamamatsu NanoZoomer Digital Pathology S360 (NDP, Hamamatsu, Herrsching am Ammersee, Germany). UC‐protruding dysplastic growths and UC‐ associated sporadic adenomas were divided into polypoid and non‐polypoid. One case with non‐polypoid elevated dysplastic lesion [24] was found in cases with UC‐PDG and another in cases with UC‐aspa.

To assess possible interobserver variations in recognizing colonic crypt phenotypes and degree of dysplasia in ulcerative colitis our group performed several validation studies [23, 25, 26, 27, 28], Based on that experience, a questionary containing a series of 30 unselected consecutive cases included in the present work was sent to two co‐workers (CLS and MV). The questionary included: (i) Crypt phenotype and (ii) Degree of cellular dysplasia. The results of this pilot study revealed a good (“substantial”) kappa agreement.

The following key features for differentiating between UP‐PDG and UC‐associated sporadic adenomas were considered:

- UP‐PDG were endoscopically lesions with irregular protruding surface and unsharped boundaries against the surrounding inflamed mucosa found in mucosal areas with active or chronic UC‐inflammation.
- UC‐associated sporadic adenomas were endoscopically well‐circumscribed lesions with or without a stalk‐like pedicle. The border against the surrounding non‐involved mucosa was usually sharp.

These two endoscopic features could be demonstrated in sections with UP‐PDG or UC‐associated sporadic adenomas including adjecent mucosa.

Unfortunately, the presence of inflammatory cells in the *lamina propria mucosa* may not be a reliable histologic parameter in differentiating between UC‐PDG and UC‐associated sporadic adenomas inasmuch as UC‐associated sporadic adenomas may disclose inflammation triggered by a variety of reasons discussed by Bilinski et al. in humans [29], and by Tanaka et al. [30] and by Karagianis et al. in experimental animals [31].

### 2.2 Histological Classification

#### 2.2.1 GACP in UC‐PDG and UC‐Aspa

**FIGURE 1:**
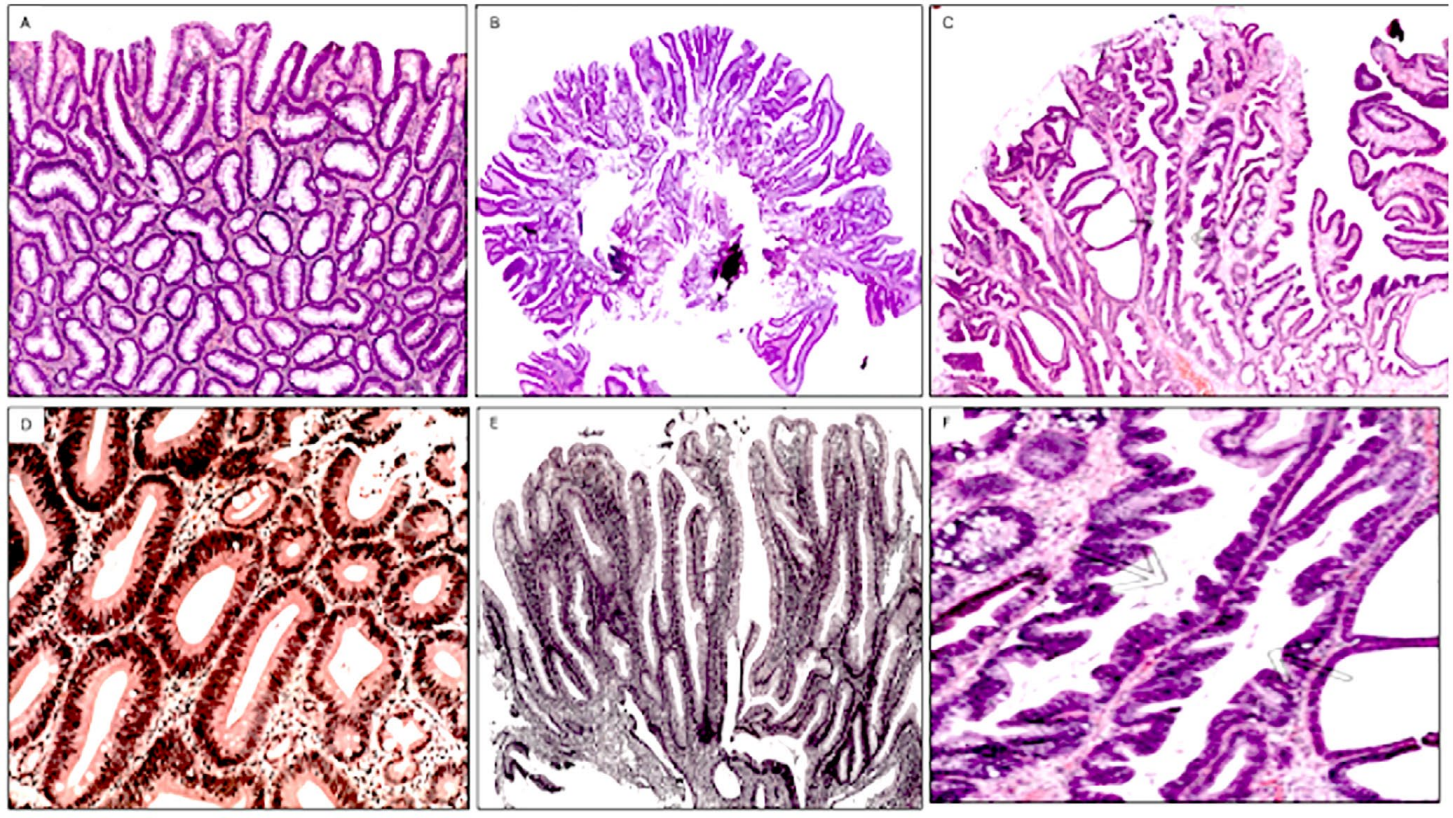
Generally accepted crypts phenotypes Upper panel. (A) Tubular crypt phenotype (protruding dysplastic growth, ulcerative colitis, (H&E, original ×10), (B) Villous crypt phenotype (protruding dysplastic growth, ulcerative colitis, H&E, original ×4), (C) Serrated crypt phenotype, sporadic adenoma in non‐colitic patient, H&E, original ×10), Lower panel panel. (A) Detail of tubular crypt phenotype (protruding dysplastic growth, ulcerative colitis, original H&E, ×20), (B) Detail of villous crypt phenotype (protruding dysplastic growth, ulcerative colitis, H&E, original ×10), (C) Detail of serrated crypt phenotype, sporadic adenoma in non‐colitic patient, (H&E, original ×20).

Long‐lasting mucosal colon inflammation leads to profound changes in the architecture of the colon crypts [8]. The histology of UC‐GACP include tubular, tubulovillous, villous [6, 8] and unclassified serrated crypt phenotypes [13, 14]. Increasing amounts of inflammatory cells enlarge the inter‐cryptal *lamina propria*, thereby contributing to the architectural distortions of the crypts [32]. UC‐PDG and UC‐aspa were histologically classified into tubular, often exhibiting irregular tubules disclosing < 25% villous formations, tubulo‐villous (having > 25% but < 75% villous formations), villous (≥ 75% villous features, epitomized by long finger or leaf‐like projecting from the surface) [12, 33] and serrated phenotypes [13, 14] (Figure 1). The latter were more recently referred to as unclassified serrated adenomas (USA) by the WHO Classification of Tumous [34]. Unclassified serrated adenomas were characterized by elongated crypts lined with spiky or scalloped epithelial folds adopting a saw‐tooth‐like mucosal architecture and cellular dysplasia. Unclassified serrated adenomas did not fulfilled the criteria of traditional serrated adenomas (TSA) as defined by the WHO Classification of Tumors [34]. TSA, also referred to as microtubular adenomas in the literture [35] were not found in this survey.

#### 2.2.2 Additional Crypt Phenotypes in GACP and UC‐Aspa

**FIGURE 2:**
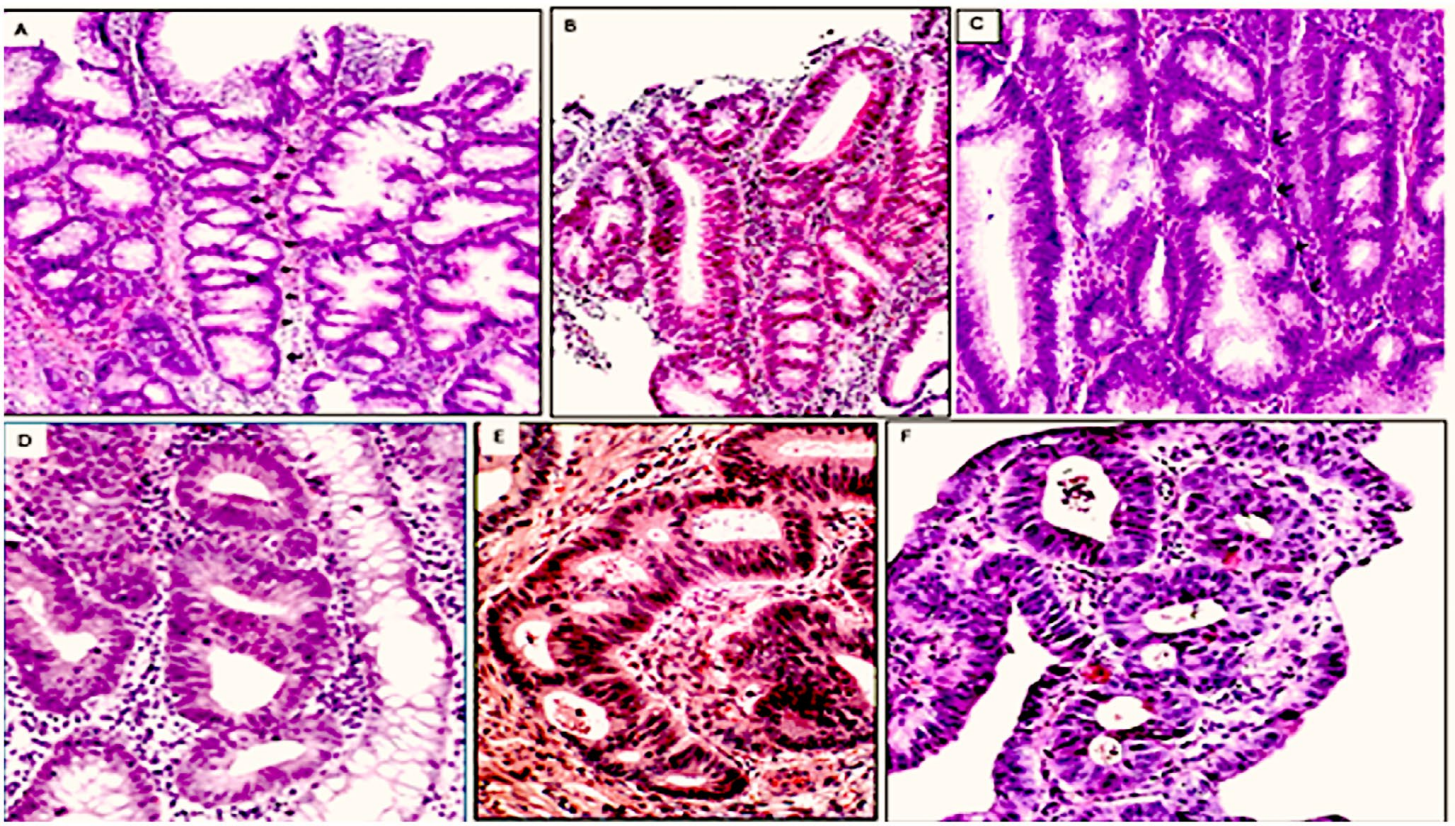
Crypts‐rings in tandem phenotype Upper panel. (A) Crypt‐rings in tandem phenotype (UC‐protruding dysplastic growth, H&E, original ×10), (B) Crypt‐rings in tandem phenotype (UC‐protruding dysplastic growth, original, H&E, ×20) (C) Crypt‐rings in tandem phenotype (UC‐associated sporadic adenoma, H&E, original ×20). Lower panel panel. (D) Detail of cryps‐rings in tandem phenotype (UC‐protruding dysplastic growth H&E, original ×20), (E) Detail of crypts‐rings in tandem phenotype (UC‐protruding dysplastic growth, H&E, original ×20), (F) Detail of crypt‐rings in tandem phenotype (UC‐associated sporadic adenoma, original ×20), Note crypts lined with high‐grade cellular dysplasia.

**FIGURE 3:**
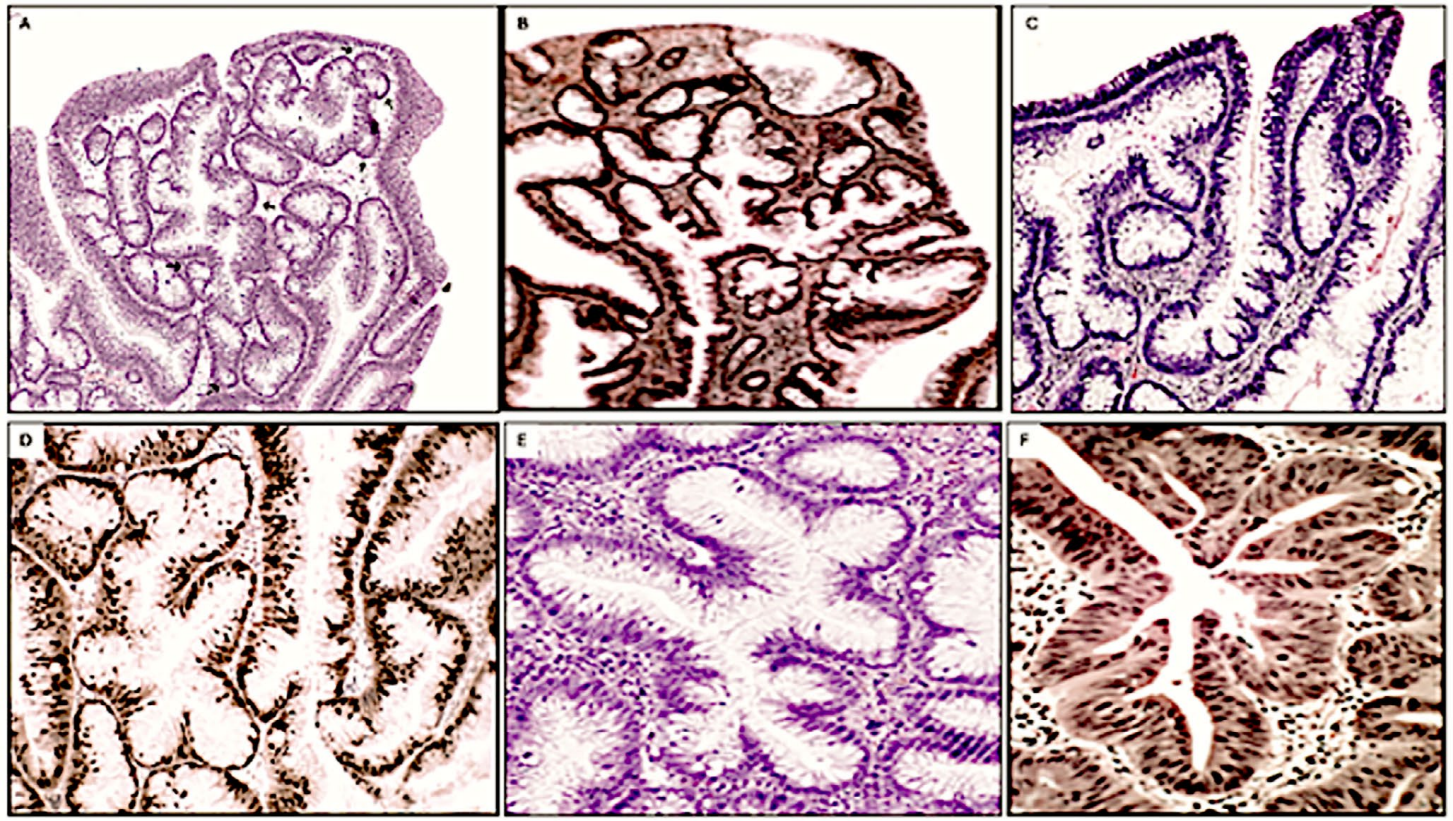
Crypts with lateral budding's phenotype Upper panel. (A) Crypts with lateral budding's phenotype (H&E, original ×10). Note the asymmetrically distributed outpouching's length the main crypt. UC‐associate sporadic adenoma. (B) Crypts with lateral budding's phenotype. Protruding dysplastic growth, ulcerative colitis (H&E, original ×10), (C) Detail of crypts with lateral budding's phenotype, Protruding dysplastic growth, ulcerative colitis (H&E, original ×20). Lower panel panel. (D) Detail of crypts with lateral budding's phenotype. UC‐associated sporadic adenoma (H&E, original ×20), (E) Detail of crypts with lateral budding's phenotype. UC‐associated sporadic adenoma (H&E, original ×20), (F) Detail of crypts with lateral budding's phenotype. Protruding dysplastic growth (ulcerative colitis, H&E, original ×20). Note crypts lined with high‐grade cellular dysplasia.

Descriptions of crypt rings in tandem (CRT) were based on observations carried out in well oriented (vertical/upright) tissue sections. CRT were found sandwiched between GACP, either as single crypts or less often, assembled in groups (Figure 2). CRT were characterized by single of multiple files of ≥three consecutive dysplastic crypt rings. Contiguous rings occasionally fused, creating a common single lumen. Cross‐cut tissue sections were disregarded as in UC some of the crypts with architectural distortions could adopt irregular ring‐formed shapes. Thus, the possibility that these interpolated crypt rings were related to adjacent crypt rings could not be determined in cross‐cut sections. Descriptions of crypts with lateral budding's (CLB's) were also based on observations carried out in well oriented (vertical/upright) tissue sections. Cross‐cut tissue sections were disregarded. CLB's were characterized by single to manifold lateral dysplastic buds bulging from one parental dysplastic crypt (Figure 3). The lateral dilatations or outpouchings were asymmetrically distributed length the crypts [22]. At the level of each bud, the wall and lumen of the crypt was wider. Small crypt outpouching's were lined predominantly with goblet cells, but larger ones revealed columnar cells with basally located hyperchromatic nuclei and occasional or no goblet cells.

#### 2.2.3 Degree of Cellular Dysplasia

The epithelium lining the crypts in GACP, in CRT, in CLB's and in UC‐aspa was found partially or totally replaced by low‐grade dysplastic cells (LGD) or high‐grade dysplastic cells (HGD) [8]. LGD was characterized by fusiform, hyperchromatic, crowded nuclei mostly confined to the basal half of the epithelium, as well as by reduced number of goblet cells. These cellular abnormalities often concurred with mild crypt‐architectural changes. HGD, was typified by and nuclear stratification with hyperchromatic, pleomorphic cells, crowded nuclei and loss of nuclear polarity surpassing the basal half of the epithelium and reaching its luminal aspect (surface). These cellular abnormalities often concurred with marked crypt‐architectural changes, such as budding or cribriform patterns.

#### 2.2.4 Ethical Approval

Ethical approval was obtained from the Ethics Committee of Friedrich‐Alexander Universität, Erlangen Nuremberg, Germany (ID no. 21–475‐Br).

#### 2.2.5 Statistical Analysis

The test of proportions was applied to compare possible statistical differences between the percentages of cases in the two independent groups (SPSS software, Sweden). The level of statistical significance was set at *p* ≤ 0.05.

## 3 Results

### 3.1 Age

Table 1 shows that the mean age in cases with UC‐PDG was 62.95 years, and in the UC‐aspa group, 60.48 years. No significant difference in the mean age was found between patients in the UC‐PDG group and in the UC‐aspa group (cfr Table 1).

**TABLE 1:** Age and gender in 100 cases with protruding dysplastic growths in UC (UC‐PDG), and in 100 cases with UC‐associated sporadic adenomas. The degree of significance by age and gender as well as the comparison between (i) UC‐PDG vs. UC‐associated sporadic adenomas, *is* also shown.

	UC‐PDG (no. 100)	UC‐associated sporadic adenomas (no. 100)
Age		
Mean (range)	62.95 (23–81)	60.48 (36–86)
Gender		
Males	73	78
Females	27	22
Males/female ratio	*p* < 0.0001*	*p* < 0.0001*

* Statistical significance at *p* < 0.05.

### 3.2 Gender

Table 1 also shows that in the UC‐PDG group, 73 were males and 27 females. In the UC‐aspa group, 78 were males and 22 females and 22 females. The male/female ratio was significantly higher in the two tested groups (*p* < 0.0001, and *p* < 0.0001).

### 3.3 UC‐GACP and UC‐Aspa

Table 2 shows the frequency of cases with GACP in the two groups investigated. It is seen that the tubular was the most frequently found crypt phenotype in the two groups, accounting for 86% (171/200) of the cases. The second most frequent was the villous phenotype; it was recorded in 8% (16/200) and the least frequent the serrated phenotype (2% or 3/200 cases). The proportion of cases with tubular crypt phenotype was significantly higher in UC‐aspa than in UC‐PDG (*p* < 0.0001). Conversely, the proportion of cases with villous phenotypes was significantly higher in UC‐PDG than in UC‐aspa (*p* < 0.0001). For tubulovillous crypt phenotypes the proportion of cases with UC‐PDG was borderline significant (*p* = 0.0522) than in those with UC‐aspa. No significant differences in the proportion of serrated crypt phenotypes were found between UC‐PDG and UC‐aspa (*p* = 0.561).

**TABLE 2:** Frequency of cases with generally accepted crypt phenotype (GACP) and additional crypt phenotypes (ACP) in ulcerative colitis (UC) and in UC‐associated sporadic adenomas (UC‐aspa). Comparison of the degree of significance between the proportion of cases in GACP and in ACP showing UC‐PDG with LGD and UC‐aspa with LGD are shown.

	UC‐PDG	UC‐aspa	Total
GAPC			
Tubular	76	95	171
Tubulovillous	8	2	10
Villous	14	2	16
Serrated	2	1	3
Total cases with GAPC	100	100	200
APC			
Crypt rings in tandem	17	16	33
Crypt with lateral budding's	61	37	98
Total cases with APC	78	53	131
GACP	Comparison between cases with UC‐PDG and UC‐aspa cases		
Tubular	*p* = 0.0001*		
Tubulovillous	*p* = 0.0522		
Villous	*p* = 0.0018*		
Serrated	*p* = 0.5617		
ACP			
Crypt rings in tandem	*p* = 0.8493		
Crypt with lateral budding's	*p* = 0.0007*		
Total ACP	*p* = 0.0002*		

* Significant at < 0.05.

### 3.4 Additional Crypt Phenotypes Interpolated Amidst GADP in UC

The proportion of cases with interpolated crypt phenotypes is shown in Table 2. It is seen that these crypt phenotypes were recorded in 66% (131/200). Of these, CRT were recorded in 25% (33/131) and CLB's in the remaining 75% (98/131). Thus, additional interpolated CLB's phenotypes amidst GACP predominated. The proportion of cases with CLB's was significantly higher than in cases with CRT (*p* = 0.0007).

### 3.5 Low‐Grade Cellular Dysplasia in Cases With GACP and With Additional Crypt Phenotypes

The frequency and proportion of cases with low‐grade cellular dysplasia in GACP and in additional crypt phenotypes is shown in Table 3. It is seen that the proportion of cases with tubular crypt phenotype with LGD was significantly higher in UC‐aspa than in UC‐PDG (*p* < 0.0001). Conversely, the proportion of cases with CLB's showing LGD was significantly higher in UC‐PDG than in UC‐aspa (*p* = 0.0395).

**TABLE 3:** Frequency of cases with generally accepted crypt phenotype (GACP) and additional crypt phenotypes (ACP) with low‐grade dysplasia (LGD) in ulcerative colitis (UC) and in UC‐associated sporadic adenomas (UC‐aspa). Comparison of the degree of significance between the proportion of cases in GACP and in ACP showing UC‐protruding dysplastic growths (UC‐PDG) with LGD and UC‐aspa with LGD is shown.

	UC‐PDG	UC‐aspa	Total
GAPC			
Tubular with LGD	62	92	154
Tubulovillous with LGD	4	2	6
Villous with LGD	5	0	5
Serrated with LGD	0	1	1
Total cases with GAPC with LGD	71	95	166
ACP			
Crypt rings in tandem with LGD	12	15	27
Crypt with lateral budding's with LGD	48	32	80
Total cases with ACP sgowing LGD	60	47	107
Comparison between the proportion of cases with crypt phenotypes	UC‐PDG vs. UC‐aspa		
GAPC			
Tubular with LGD	*p* < 0.0001*		
Tubulovillous with LGD	*p* = 0.4083		
Villous with LGD	*p* = 0.0239*		
Serrated with LGD	*p* = 0.3173		
Total GAPC	*p* < 0.0001*		
ACP			
Crypt rings tandem with LGD	*p* = 0.5358		
Crypt s lateral budding's with LGD	*p* = 0.0212*		
Total ACP	*p* = 0.0660		

* Significant at < 0.05.

### 3.6 High‐Grade Cellular Dysplasia in Cases With GACP and With Additional Crypt Phenotypes

Table 4 shows that HGD was recorded in 17% (34/200) of cases with GACP and in 18% (24/131) of the cases with additional crypt phenotypes. HGD predominated (50%) in cases with tubular crypt phenotypes (17/34). The proportion of cases with tubular, tubulovillous and villous crypt phenotypes with HGD were significantly higher in UC‐PDG than in UC‐aspa (*p* = 0.0054, *p* = 0.0439, and *p* = 0.0303, respectively).

**TABLE 4:** Frequency of cases with generally accepted crypt phenotype (GACP) and additional crypt phenotypes (ACP) with high‐grade dysplasia (HGD) in ulcerative colitis (UC) and in UC‐associated sporadic adenomas (UC‐aspa). Comparison of the degree of significance between the proportion of cases in GACP and in ACP showing UC‐PDG with LGD and UC‐aspa with HGD is shown.

	UC‐PDG	UC‐aspa	Total
GAPC			
Tubular with HGD	14	3	17
Tubulovillous with HGD	4	0	4
Villous with HGD	9	2	11
Serrated with HGD	2	0	2
Total cases with generally accepted crypt phenotypes with HGD	29	5	34
ACP			
Crypt rings tandem with HGD	5	1	6
Crypt s lateral budding's with HGD	13	5	18
Total cases with ACP showing HGD	18	6	24
Comparison between the proportion of cases with crypt phenotypes	UC‐PDG vs. UC‐aspa		
GAPC			
Tubular with HGD	*p* = 0.0054		
Tubulovillous with HGD	*p* = 0.0439*		
Villous with HGD	*p* = 0.0303*		
Serrated with HGD	*p* = 0.1563		
Total GAPC with HGD	*p* < 0.0001*		
ACP			
Crypt rings tandem with HGD	*p* = 0.0981		
Crypt s lateral budding's with HGD	*p* = 0.0486*		
Total ACP with HGD	*p* = 0.0092*		

* Significant at < 0.05.

Crypt phenotypes cellular HGD accounted for 57% (29/34) of the total number of additional UC‐cases with HGD (Table 3). The proportion of cases with CLB's with cellular HGD predominated (75% or 18/24) (Table 4). The proportion of cases with CLB's was significantly higher in UC‐PDG than in UC‐aspa (*p* = 0.0486).

## 4 Discussion

Several authors reported no significant difference in the histological characteristics between UC‐protruding dysplastic lesions and US‐associated sporadic adenomas [15, 16, 17, 18, 19]. For this investigation it was hypothesized that in addition to the four crypt phenotypes present in GACP, the presence of the two additional crypt phenotypes (i.e., CRT and CLB's, accounting for 66% or 2/3 of the 200 cases) could increase the possibility to histologically discriminate between UC‐PDG and UC‐aspa. We found significantly higher proportion of cases with villous phenotype without or with HGD, with CLB's without or with HGD, and with tubulovillous phenotype with HGD in UC‐PDG than in UC‐aspa. Moreover, the total proportion of cases with UC‐PDG with HGD as well as the total proportion of ACP cases with HGD were significantly higher in UC‐PDG than in UC‐aspa.

It is generally accepted that HGD evolving in UC‐PDG (i.e., in areas with chronic UC‐inflammation) carries a higher risk of progressing towards colorectal cancer than HGD detected in UC‐aspa (i.e., growing in areas without inflammation) [1, 8], The present histological findings substantiate that clinical experience.

**FIGURE 4:**
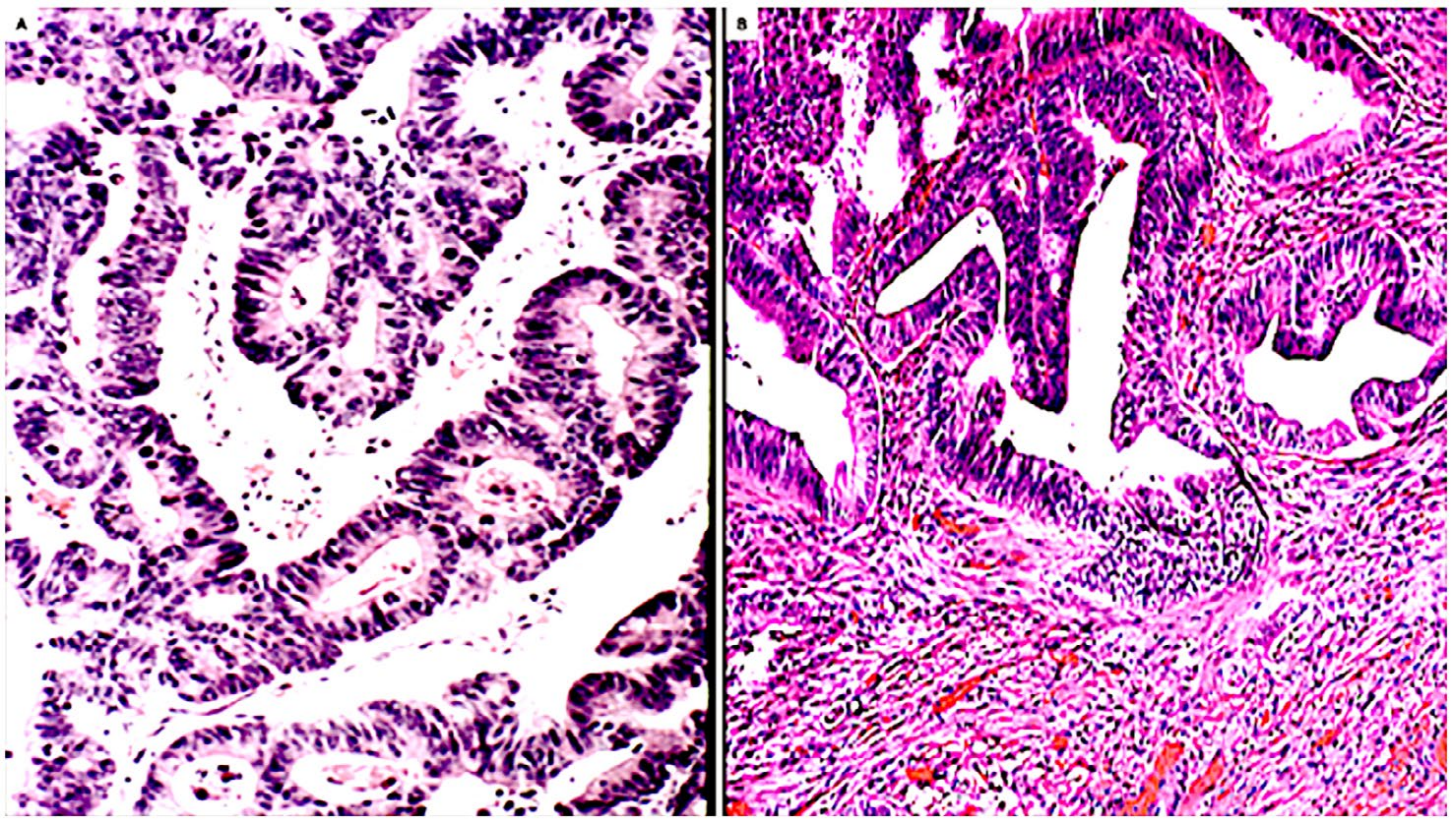
Invasive colon adenocarcinoma Neoplastic glands portraying ring‐shapes in tandem. (H&E, colon adenocarcinoma, original ×20) Neoplastic glands disclosing lateral budding's (H&E, colon adenocarcinomas original ×10).

The possible participation of CRT and CLB's in colorectal carcinogenesis remains elusive. Nonetheless, it should be mentioned that previous histologic studies of invasive colorectal carcinoma revealed six predominant histological phenotypes: tubular, serrated‐microcystic, villous, fenestrated, signet ring cell, and undifferentiated [36]. More recently we observed occasional cases of colon adenocarcinomas depicting rings in tandem‐neoplastic glands (Figure 4A) or with neoplastic glands portraying lateral budding's (Figure 4B). Whether or not these configurations originated in adenomatous lesions with dysplastic CRT and with dysplastic CLB's, respectively, remains speculative.

## 5 Conclusion

The different number of cases disclosing the above‐mentioned histological parameters in each group permitted to discriminate between UC‐PDG and UC‐asta.

In closing it should be mentioned that crypts in asymmetric fission in UC were recently identified by the aid of artificial intelligence (AI) [37]. This cutting‐edge technique opens new vistas for future studies on the possible AI identification of GAPC, CRT and CLB's in UC‐PDG and in UC‐associated sporadic adenomas in digitalized colon biopsies from patients with UC.

## Funding

The authors have nothing to report.

## Conflicts of Interest

The authors declare no conflicts of interest.

## Data Availability

Data sharing not applicable‐no new data generated, or the article describes entirely theoretical research.
